# Cleavage of Phage DNA by the *Streptococcus thermophilus* CRISPR3-Cas System

**DOI:** 10.1371/journal.pone.0040913

**Published:** 2012-07-20

**Authors:** Alfonso H. Magadán, Marie-Ève Dupuis, Manuela Villion, Sylvain Moineau

**Affiliations:** 1 Département de Biochimie, de Microbiologie et Bio-Informatiques, Faculté des Sciences et de Génie, Groupe de Recherche en Ecologie Buccale, Université Laval, Québec, Canada; 2 Félix d’Hérelle Reference Center for Bacterial Viruses, Université Laval, Québec, Canada; University of Massachusetts Medical School, United States of America

## Abstract

*Streptococcus thermophilus*, similar to other *Bacteria* and *Archaea*, has developed defense mechanisms to protect cells against invasion by foreign nucleic acids, such as virus infections and plasmid transformations. One defense system recently described in these organisms is the CRISPR-Cas system (Clustered Regularly Interspaced Short Palindromic Repeats loci coupled to CRISPR-associated genes). Two *S. thermophilus* CRISPR-Cas systems, CRISPR1-Cas and CRISPR3-Cas, have been shown to actively block phage infection. The CRISPR1-Cas system interferes by cleaving foreign dsDNA entering the cell in a length-specific and orientation-dependant manner. Here, we show that the *S. thermophilus* CRISPR3-Cas system acts by cleaving phage dsDNA genomes at the same specific position inside the targeted protospacer as observed with the CRISPR1-Cas system. Only one cleavage site was observed in all tested strains. Moreover, we observed that the CRISPR1-Cas and CRISPR3-Cas systems are compatible and, when both systems are present within the same cell, provide increased resistance against phage infection by both cleaving the invading dsDNA. We also determined that overall phage resistance efficiency is correlated to the total number of newly acquired spacers in both CRISPR loci.

## Introduction

Clustered regularly interspaced short palindromic repeats (CRISPR) along with *cas* genes have been observed in the genomes of various *Archaea* and *Bacteria* [1]–[3]. CRISPR loci are composed of short direct DNA repeats interspersed with short non-repetitive nucleotides called spacers. These systems are encoded by operons possessing extraordinarily diverse architectures for both the *cas* genes and the spacer content. Accordingly, CRISPR-Cas systems are currently classified into three major types (I, II, and III) and additional subtypes [4]. One of the key roles of CRISPR-Cas systems is to interfere with invading foreign nucleic acids such as viruses and plasmids [5]–[7].

A few functional stages are needed for a CRISPR-Cas system to play its defensive role [8]. In the acquisition step, a new repeat-spacer unit is added at the 5′ end of the CRISPR locus, in which the spacer comes from the invading nucleic acid. Spacer acquisition likely goes through a recognition process involving specific sequences known as PAMs (Protospacer-Adjacent Motifs), which flank the invading protospacer sequence [9]. Another step involves the biogenesis of small RNAs. The CRISPR locus is transcribed from a leader/promoter region into a long RNA (pre-crRNA) containing the full set of repeat-spacer units and is subsequently processed into mature small RNAs (crRNA) containing the spacer and parts of adjacent repeats at its 5′ and/or 3′ extremities [10], [11]. The processing of the pre-crRNA varies according to the type of CRISPR-Cas system. Finally, these short CRISPR-derived RNAs assemble with Cas proteins into large surveillance complexes that target and cleave the invading genetic material [7].

**Figure 1:**
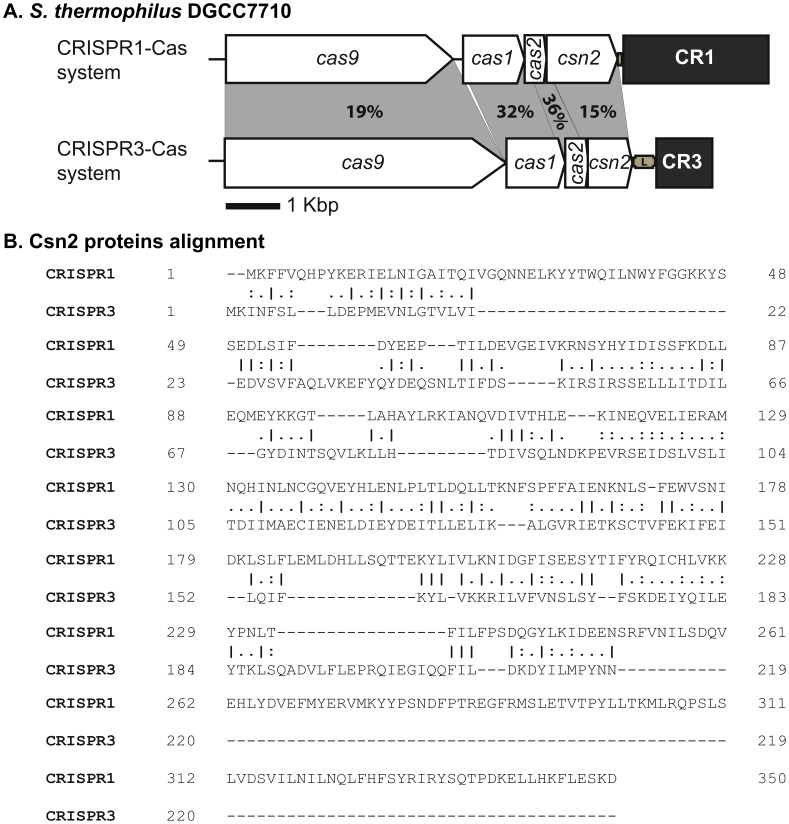
Architecture of CRISPR1-Cas and CRISPR3-Cas systems of *S. thermophilus* DGCC7710. **A**. CRISPR loci are represented by grey boxes. The percentages of amino acid identity between the Cas protein sequences are indicated in the grey shading. The percentages of identity were calculated by dividing the number of identical residues per the length of the alignment (i.e., the higher protein size). **B**. Csn2 amino acid alignment of CRISPR1-Cas and CRISPR3-Cas systems. Bars, dots, and hyphens emphase identical amino acids and gaps, respectively. Numbers at the right side are the positions on the related protein sequence. EMBOSS Needle (from the European Bioinformatics Institute) was used to align protein sequences (http://www.ebi.ac.uk/Tools/psa/emboss_needle/).

While CRISPR-Cas systems have been described in several species, only a few have been studied in detail, including two type II systems in *S. thermophilus*: CRISPR1-Cas and CRISPR3-Cas [8], [12]–[14]. Type II systems (exclusive to *Bacteria*) are the simplest CRISPR-Cas systems in terms of the number of associated genes. They are recognized by the presence of four genes including the signature gene *cas9*, in addition to the universal *cas1* and *cas2* genes. Two subtypes can be distinguished based on the fourth gene: subtype II-A contains a *csn2-like* gene whereas subtype II-B contains a *cas4-like* gene [4]. It has been shown that inactivating the *cas9* gene of the CRISPR1-Cas system eliminates the phage resistance phenotype [8], [15] whereas when the *csn2*-like gene is inactivated, spacer acquisition is no longer possible [8], [15].

Another interesting feature of type II systems is the biogenesis of crRNAs. A small RNA, called tracrRNA (trans-acting CRISPR RNA), is produced that is partially complementary to the CRISPR repeat. This tracrRNA hybridizes to the repeat and the host protein RNase III processes the RNA duplex [11]. Cas9 also acts during that process although its precise role is still undefined [11], [16]. Finally, it was recently shown that the interference process of the CRISPR1-Cas system of *S. thermophilus* is due to the cleavage of the foreign dsDNA within the protospacer at a specific distance from the PAM and in an orientation-dependent manner [15].

Overall, little information is available about CRISPR3-Cas system. *In silico* analyses first suggested that this system was active in some *S. thermophilus* strains [13]. These bioinformatic data were later confirmed through the generation of bacteriophage-insensitive mutants (BIMs) [13], [14]. Recently, it was shown that the streptococcal CRISPR3-Cas system is functional on its own and could interfere with plasmid transformation when transferred into *E. coli* [16]. We further investigated the acquisition and interference stages of the *S. thermophilus* CRISPR3-Cas system in its original host.

## Results and Discussion

### Analysis of Type II CRISPR-Cas Systems

*S. thermophilus* DGCC7710 contains two active type II CRISPR-Cas systems: CRISPR1-Cas and CRISPR3-Cas [8], [12]–[14], and at first look, their Cas-cluster organization seems to be similar each other [Figure 1A] and similar to the type II CRISPR systems of the strain *S. thermophilus* LMD-9. A sequence analysis of the latter strain performed by Deltcheva *et al.* [11] showed that the tracrRNA is located at different positions. In CRISPR1, it is in the sense orientation and located between *cas9* and *cas1* genes while the tracrRNA of CRISPR3 is an antisense orientation and upstream of the *cas*-cluster [11]. Both type II systems of *S. thermophilus* DGCC7710 currently belong to CRISPR-Cas subtype II-A [4], but a closer look at the Cas proteins showed important differences. The Cas proteins have limited identity to each other: 19%, 32%, 36% and 15% for Cas9, Cas1, Cas2, and Csn2 proteins, respectively [Figure 1A]. There is also a significant size difference between the signature Cas9 and Csn2 proteins. Cas9 of CRISPR3 has 1388 amino acids (aa) as compared to 1121 aa for the Cas9 of CRISPR1. Similarly, Csn2 of CRISPR3 is much shorter with 219 aa as compared to the 350-aa Csn2 of CRISPR1 [Figure 1A].

### Analysis of Isolated BIMs

In order to study the CRISPR3-Cas system, virulent phage 2972 [17] and the phage-sensitive host strain *S. thermophilus* DGCC7710 were selected for phage challenge assays [12], [13]. The CRISPR1 and the CRISPR3 loci of wild-type DGCC7710 contain 32 and 12 spacers, respectively. Of all the spacers, only one shares homology with the 2972 phage genome. Strain DGCC7710’s 6^th^ CRISPR3 spacer perfectly matches a region of the phage, but no interference was observed. Sequence analysis of phage 2972 genome revealed the absence of a PAM motif flanking this region.

Overall, 320 *S. thermophilus* DGCC7710 BIMs were independently generated after challenging the wild-type strain with phage 2972 (Table 1). Because *S. thermophilus* DGCC7710 contains four CRISPR systems (CRISPR2/subtype III, CRISPR4/subtype I, CRISPR1 and CRISPR3/subtype II), all four CRISPR loci were analyzed in each BIM. As previously observed [13], none of the BIMs acquired new spacers in their CRISPR2 or CRISPR4 loci. For this phage-host pair, approximately 10% (30/320) of the BIMs analyzed acquired a new spacer in the CRISPR3 locus (Table 1). Two BIMs acquired a distinct spacer in both type II CRISPR loci, whereas the remaining BIMs (288/320) acquired a spacer in only the CRISPR1 locus. Of note, two BIMs also experienced spacer deletions in the CRISPR1 locus (Table 1) [12]. Multiple spacer insertions were also observed in 18 BIMs (5.6%), most of them involving the CRISPR1 locus (17/18), and only one BIM acquired two new spacers in the CRISPR3 locus (Table 1). The results obtained using *S. thermophilus* DGCC7710 and phage 2972 supports the idea of increased spacer acquisition activity for the CRISPR1 locus compared to the CRISPR3 locus [13].

**Table 1:** Characteristics of *S. thermophilus* DGCC7710 BIMs.

Bacteriophage-insensitive mutants (%)	
N° of BIMs	320
This study	292/320 (91.3)
Deveau *et al.* (2008) [12]	26/320 (8.1)
Garneau *et al.* (2010) [15]	2/320 (0.6)
CRISPR loci of BIMs	
CRISPR1	288/320 (90.0)
CRISPR3	30/320 (9.4)
CRISPR1-CRISPR3	2/320 (0.6)
N° of deletions*	2/320 (0.6)
N° of single insertions	302/320 (94.4)
N° of multiple insertions	18/320 (5.6)
CRISPR1 double	14/18 (77.8)
CRISPR3 double	1/18 (5.6)
CRISPR1-CRISPR3	2/18 (11.1)
Quadruple*	1/18 (5.6)
**Bacterial spacers**	
N° of identified spacers	338
CRISPR1	307/338 (90.8)
CRISPR3	31/338 (9.2)
N° of different spacers	163/338 (48.2)
CRISPR1	139/307 (45.3)
CRISPR3	24/31 (77.4)
Spacers length CRISPR1	
<30 nt <	31/307 (10.1)
= 30 nt	276/307 (89.9)
Spacers length CRISPR3	
<30 nt <	1/31 (3.2)
= 30 nt	30/31 (96.8)
**Phage protospacers**	
N° of different CRISPR1	139
Coding strand	98/139 (70.5)
Non-coding strand	41/139 (29.5)
N° of different CRISPR3	22
Coding strand	14/22 (63.6)
Non-coding strand	8/22 (36.4)

* Only in CRISPR1.

The analyses of CRISPR1 BIMs were previously reported in detailed elsewhere [12]. We observed the same general conclusions in this study, such as polarized spacer insertion at the 5′-end of the locus, mostly single spacer acquisition in the BIMs, spacer size of 30-nt in 90% of cases, and the importance of PAM motifs. Therefore, we will focus here on CRISPR3 BIMs.

**Table 2:** List of spacers found in *S. thermophilus* CRISPR3 loci.

Spacer^a^	Position^b^	Length	Sequence (5′→3′)	PAM^c^	ORF; function^d^
S61	2095–2066	30	CAAAATATCTCTATCGTAGAATTTGCCCTT	GGGCG	ORF3; large terminase
S62	3181–3152	30	TCTTGAATGCGTGGGGCTTGTCTCAATTTA	TGGTG	ORF5; portal protein
S63	3713–3742	30	TACGGAATATTTAAATAATATTGATGGCAT	TGGTG	ORF5; portal protein
S64	6300–6329	30	TATCAAAGCAGGTACCCTCGTAGCAGGTGA	TGGCG	ORF8; capsid protein
S65	6515–6544	30	CACTCGTTAAATTTATCTCTAAGAAATAAG	AGGAG	ORF8; capsid protein
S66	6978–7007	30	TGCTACTGGTAAAATTGCATTTACGAGCGA	TGGAG	ORF9; capsid protein
S67	13,754–13,783	30	CAGGGTTGACCATGTTCAGCGCAGTAGCGA	TGGCG	ORF18; MTP
S68	17,665–17,636	30	CTCAACGGCACGCGATCTTATGTCAAGCGC	TGGTG	ORF20; RBP
S69	21,076–21,105	30	TCAGCGGGTATCAACCATTGCTAAGGAATT	AGGTG	ORF20; RBP
S70	21,346–21,375	30	TAACCCAGACATGAATGTCATTCGCTATGT	AGGAG	ORF20; RBP
S71	21,670–21,699	30	TTTCTCTTTTTCAGCGCAGTTTAATGGTTC	TGGTG	ORF21; tail protein
S72	23,182–23,211	30	TCTGACGGTTAGATATAATTTTACTGGTAA	TGGGG	ORF21; tail protein
S73	23,329–23,300	30	TATTTGGGCGTGAGTATTGTAACTTCCGCT	AGGGG	ORF21; tail protein
S74	24,038–24,067	30	CATGAGGGCAACTTGGACGATTGATAAGGT	TGGCG	ORF24; unknown
S75	24,325–24,354	30	AAACACAGATGTTTTAGACCATGCGCAGAA	GGGAG	ORF24; unknown
S76	25,297–25,326	30	TCAGTATAAACTAAGTGAACGCAAACAAAG	CGGTG	ORF26–ORF27**
S77	26,729–26,758	30	TAAACCGTTCTTCAATCCGTAGCCACACCC	TGGTG	ORF32–ORF33**
S78	26,917–26,946	30	CCATGAAAACATTTAAAATTACAACAATTA	GGGAG	ORF32–ORF33**
S79	27,975–28,004	30	TGCTCGACTTGTTAAAAAAACTACTGAAGA	TGGCG	ORF34; SSAP
S80	28,087–28,058	30	TACACCCCTTTCGCTCATCTAAGCGGTTTT	TGGCG	ORF34; SSAP
S81	29,120–29,091	30	TATTAACACCTTTTAGTGACCATTCACGGT	CGGCG	ORF35; helicase
S82	29,121–29,091	31	TTATTAACACCTTTTAGTGACCATTCACGGT	CGGCG	ORF35; helicase
S83	30,033–30,004	30	CCACACCCTCCGAATGTCGTTTTCAGTCAT	TGGTG	ORF37; replication
S84	34,662–34,633	30	CACTTTCCTTTTCAAGACCTAGATCACCTT	TGGTG	ORF44; unknown
S85*	16,681–16,652	30	TGTTTCAAGGTTTCGGGTCCAAGTATCATT	ATAGAAA	ORF20; RBP
S86*	29,923–29,894	30	TTATGGAGATGGTTGATTACGCAATCAACT	TTAGAAT	ORF37; replication

a These spacers from CRISPR1 locus are specified (*) since they were acquired in BIMs having double spacer acquisitions in this study.

b The position of the protospacer in the phage 2972 genome.

c PAMs are 5′-NNAGAAW-3′ and 5′-NGGNG-3′ for CRISPR1 and CRISPR3 protospacers, respectively. Consensus nucleotides are underlined.

d Spacers found in intergenic regions are also specified (**).

MTP: major tail protein; RBP: Receptor Binding Protein; SSAP: Single Strand Annealing Protein.

Analysis of the 31 spacers acquired by the CRISPR3 system revealed 24 unique spacers (Table 2). Spacer S79 was detected three times, while five spacers (S61, S62, S63, S69, and S80) were observed twice. It is unclear if these six spacers were acquired more frequently or if the increased frequency was due to multiplication of the ancestor. Of note, all CRISPR3 spacers isolated were 30 nucleotides long, with the exception of S82 (31 nucleotides) (Table 2). Analysis of the corresponding protospacers in the phage genome revealed the presence of a correct PAM motif (NGGNG) at the 3′-end [12], [13].

### Phage Interference

The level of phage protection (efficiency of plaquing, EOP) provided by the addition of a new repeat-spacer unit in the CRISPR3 locus was determined using plaque assays (Table 3). Overall, the EOPs of phage 2972 (about 10^−4^ to 10^−6^) were similar to those reported for CRISPR1 BIMs [8], [12]. The EOP of 2972 on the previously described BIM S4 [12], [15], which has acquired a new spacer into CRISPR1, was also about 10^−6^ (Table 1). However, the phage resistance levels increased significantly when a second spacer was acquired either in the CRISPR3 (Table 3, BIM S61/S78) or in the CRISPR1 locus (Table 3, BIMs S61/S85 and S75/S86). All levels of phage resistance were comparable to previous reports the BIM with multiple spacer acquisitions into CRISPR1 [12], [15]. Interestingly, the level of phage resistance was similar when comparing a BIM that had acquired two spacers in CRISPR3 and a BIM that had acquired a new spacer in each of CRISPR1 and CRISPR3 (Table 3). These data suggest that the CRISPR1-Cas and CRISPR3-Cas systems are compatible within the same strain. Moreover, it shows that even if the acquisition stage is more efficient for CRISPR1, the interference stage is similar for both CRISPR-Cas systems in this *S. thermophilus* strain.

**Table 3:** *S. thermophilus* BIMs used in the study and details on cleavage and EOP.

Strain name	Other name^a^	Spacer names	CRISPR loci	Sequence (5′ → 3′)^c^	PAM^d^	EOP values^e^
DGCC7710_Φ2972_^+S4^	BIM S4 b	S4	CRISPR1	CTCAGTCGTT 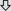 ACTGGTGAACCAGTTTC** 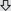 **AAT	TGAGAAA	(6.7±5.4)×10^−6^
DGCC7710_Φ2972_^+S85^	BIM S85	S85	CRISPR1	TGTTTCAAGGTTTCGGGTCCAAGTATC 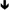 ATT	ATAGAAA	(3.0±2.0)×10^−6^
DGCC7710_Φ2972_^+S73^	BIM S73	S73	CRISPR3	TATTTGGGCGTGAGTATTGTAACTTCC 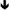 GCT	AGGGG	(3.6±1.7)×10^−4^
DGCC7710_Φ2972_^+S77^	BIM S77	S77	CRISPR3	TAAACCGTTCTTCAATCCGTAGCCACA 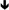 CCC	TGGTG	(9.6±0.8)×10^−6^
DGCC7710_Φ2972_^+S79^	BIM S79	S79	CRISPR3	TGCTCGACTTGTTAAAAAAACTACTGA 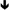 AGA	TGGCG	(6.9±4.0)×10^−6^
DGCC7710_Φ2972_^+S81^	BIM S81	S81	CRISPR3	TATTAACACCTTTTAGTGACCATTCAC 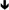 GGT	CGGCG	(1.1±0.4)×10^−5^
DGCC7710_Φ2972_^+S82^	BIM S82	S82	CRISPR3	TTATTAACACCTTTTAGTGACCATTCAC 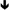 GGT	CGGCG	(9.4±3.0)×10^−6^
DGCC7710_Φ2972_^+S61+S85^	BIM S61/S85	S61	CRISPR3	CAAAATATCTCTATCGTAGAATTTGCC 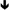 CTT	GGGCG	<10^−8^
		S85	CRISPR1	TGTTTCAAGGTTTCGGGTCCAAGTATC 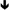 ATT	ATAGAAA	
DGCC7710_Φ2972_^+S75+S86^	BIM S75/S86	S75	CRISPR3	AAACACAGATGTTTTAGACCATGCGCA 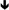 GAA	GGGAG	<10^−8^
		S86	CRISPR1	TTATGGAGATGGTTGATTACGCAATCA 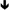 ACT	TTAGAAT	
DGCC7710_Φ2972_^+S61+S78^	BIM S61/S78	S61	CRISPR3	CAAAATATCTCTATCGTAGAATTTGCC 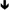 CTT	GGGCG	<10^−8^
		S78	CRISPR3	CCATGAAAACATTTAAAATTACAACAA 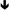 TTA	GGGAG	

a The other names are used in the text to help the reading.

b BIM S4 was characterized elsewhere [12], [15] and used here as a control.

c Experimentally proved cleavage site are symbolized with plain arrows, previously published [12], [15] cleavage sites are represented by clear arrows, whereas not experimentally determined cleavage sites are represented by grey arrows.

d PAMs are 5′-NNAGAAW-3′ and 5′-NGGNG-3′ for CRISPR1 and CRISPR3 protospacers, respectively. Consensus nucleotides are underlined.

e EOPs are the mean of at least three independent assays.

### The CRISPR3-Cas System Cuts Viral DNA within the Protospacer

We previously showed that the CRISPR1-Cas system of *S. thermophilus* cleaves phage and plasmid DNA within the protospacer [15]. Using the same methodology we investigated the cleavage site of the CRISPR3-Cas system in several BIMs (Table 3). BIMs S61/S78 and S61/S85 were selected because they acquired two different spacers after challenge with phage 2972. BIM S61/S78 acquired S61 and S78 both in the CRISPR3 locus (with S78 as the last acquired spacer) whereas BIM S61/S85 acquired S61 in its CRISPR3 locus and S85 in its CRISPR1 locus (Table 3). Phage 2972 EOP values were similar on both BIMs (<10^−8^).

**Figure 2:**
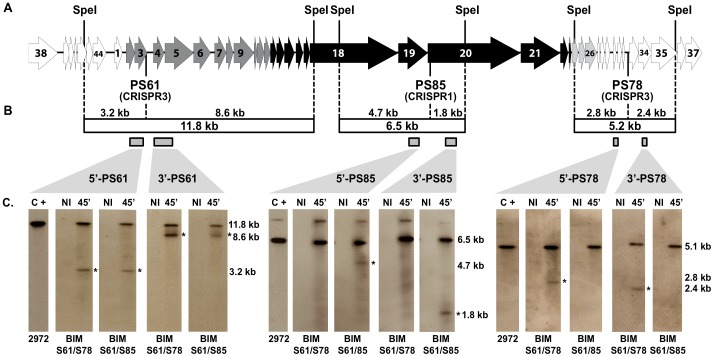
Simultaneous cleavage of phage DNA by CRISPR1-Cas and CRISPR3-Cas systems of *S. thermophilus* BIMs. **A**. Representation of the phage 2972 genome. Arrows symbolize the open reading frames (ORFs) as identified previously [18] and colors indicate associated transcriptional modules [24]. Three protospacers (PS61, PS78 and PS85) are positioned below the genome and the CRISPR-Cas systems that have acquired the respective spacers are specified (CRISPR1 or CRISPR3). **B**. The fragment sizes obtained from restriction and/or CRISPR digestion are specified. Probes used in the Southern blot assays are also indicated and details of the primers used are presented in Supplemental Table S1. **C**. Comparison by Southern blot assays of CRISPR-Cas cleavage occurring in the two selected BIMs S61/S78, and S61/S85, which share the S61 spacer (CRISPR3), but not a second spacer (CRISPR3 and CRISPR1, respectively). The Southern profiles show that independent cleavage of CRISPR1-Cas (PS85) and CRISPR3-Cas (PS61 and PS78) systems can occur simultaneously, when infected by phage 2972. Corresponding cleavage bands are identified with asterisks, and their sizes are specified. The two BIMs were infected with phage 2972 for 45 minutes before total DNA was extracted. Five micrograms of SpeI-restricted DNA was then loaded in each lane. NI, non-infected strain. C+, 10 ng of 2972 phage DNA digested with SpeI.

Phage infection assays were conducted in liquid broth and phage-infected cell samples were taken over time. Total cell DNA was extracted from non-infected and phage-infected samples, digested with SpeI endonuclease to obtain smaller phage DNA fragments. Then, restricted DNA samples were submitted to electrophoresis on agarose gels and Southern hybridization experiments were performed using six probes targeting adjacent regions to the protospacers PS61, PS78, or PS85 (Figure 2). Protospacer PS61 exists in the phage genome within an 11.8 kb SpeI-fragment (Figure 2A). In phage-infected BIMs S61/S78 and S61/S85, this phage DNA fragment was cleaved into two fragments of 3.2 kb and 8.6 kb and, based on the probes used (5′-PS61 and 3′-PS61, respectively), the cleavage site was in the vicinity of protospacer PS61 (Figure 2C). Of note, the uncut 11.8 kb fragment was also observed in these phage-infected BIMs, as was observed for CRISPR1 [15].

Protospacer PS78 is located on a 5.2 kb SpeI-fragment in the genome of phage 2972 (Figure 2A). In phage-infected BIM S61/S78, this dsDNA fragment was cleaved into two fragments of 2.8 kb and 2.4 kb and the cleavage site was also in the vicinity of the protospacer PS78 (Figure 2C). As expected, in phage-infected BIM S61/S85, the protospacer PS78 was not cleaved. These data indicate that in the phage-infected BIM S61/S78, the phage dsDNA genome was cleaved twice during the infection assay, in proximity of both protospacers (PS61 and PS78), thereby explaining the increase in phage resistance (<10^−8^).

The protospacer PS85 (acquired as a spacer in the CRISPR1 locus) is located on a 6.5 kb SpeI-fragment on the genome of phage 2972 (Figure 2A). In phage-infected BIM S61/S78, this dsDNA fragment was not cleaved. However, in phage-infected BIM S61/S85, this fragment was cleaved into two fragments of 4.7 kb and 1.8 kb and the cleavage site was again in the vicinity of the protospacer PS85 (Figure 2C). These data indicate that in the phage-infected BIM S61/S85, the phage dsDNA genome was cleaved twice in proximity of both protospacers (PS61 and PS85), thereby indicating that both CRISPR1-Cas and CRISPR3-Cas systems were compatible and acting together to increase phage resistance.

Taken altogether, the CRISPR3-Cas system also targets *in vivo* phage DNA, as does the CRISPR1-Cas system [15]. The relative quantity of cleaved phage DNA fragments in the infected BIMs was similar for both systems, confirming that their interference activity is comparable.

**Figure 3:**
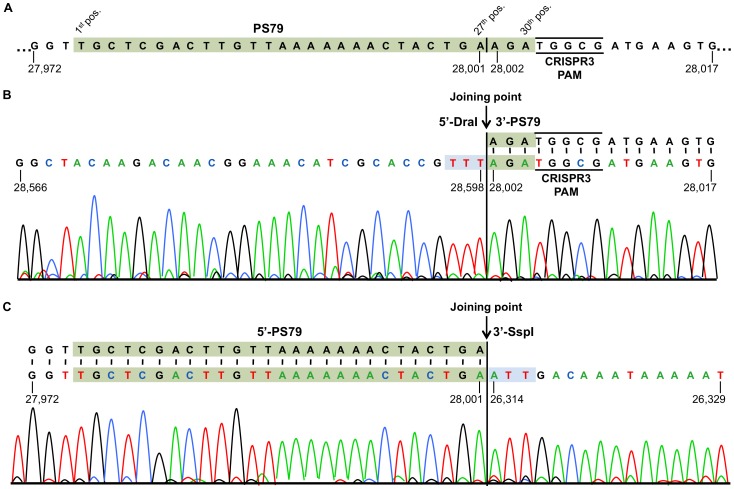
Determination of the cleavage site in 2972 phage genome by the BIM S79 CRISPR3-Cas system. Nucleotide positions of the phage genome sequence are given for each fragment and the CRISPR3 PAM is underlined. **A**. Phage 2972 genome sequence showing the protospacer PS79 and surrounding regions. The protospacer’s nucleotide positions (1^st^, 27^th^, and 30^th^) are indicated by a vertical black line to highlight the distal dsDNA cleavage position occurring after the 27^th^ nucleotide, at the extremity near the PAM (5′-NGGNG-3′). **B**. Sequencing chromatograms of inverse PCR reactions following intramolecular ligation of the 3′-PS79 end, produced by CRISPR3 cleavage, and the blunt–end, produced by DraI restriction. **C**. Sequencing chromatograms of inverse PCR reactions following intramolecular ligation of the 5′-PS79 end, produced by CRISPR3 cleavage, and the blunt–end, produced by SspI restriction. In both B and C, the joining point corresponding to the ligation of both extremities is indicated.

### Determination of the Cleavage Site

The CRISPR3-Cas cleavage site was determined in eight different BIMs (Table 3) using total DNA extracted from BIM cells infected with phage 2972. Total DNA was then digested *in vitro* by restriction endonucleases producing blunt ends (DraI, EcoRV, MlyI, ScaI, and/or SspI). The selected endonucleases cut upstream and downstream of each protospacer to allow intra-molecular ligation of fragments comprising the cleaved protospacer at one end and the blunt end at the other. We previously showed that the CRISPR1-Cas system generates blunt ends while cleaving invading phage and plasmid dsDNA [15]. The ligated molecules were then amplified by PCR and sequenced. PCR products were obtained for every tested BIM. Sequence analysis of PCR products revealed that the phage DNA had been cleaved within the selected protospacers. An example of the nucleotide sequence obtained following ligation of the cleaved protospacer PS79 to SspI-or DraI-ends is shown in Figure 3. For all analyzed cleaved protospacers, a single cleavage site was located three nucleotides upstream of the CRISPR3 PAM (Table 3, see plain arrows). Of note, the cleavage site within PS82, a 31 nucleotide-long version of PS81 (30 nucleotides), was at the exact same position as PS81, indicating that the cleavage likely implicates the PAM sequence.

The cleavage site determined for the CRISPR3-Cas system is at the same position as observed for the CRISPR1-Cas system [15]. However, contrary to the CRISPR1-Cas system, we did not observe a second cleavage site (19^th^ or 20^th^ nucleotide upstream of the PAM) targeting the coding strand (see BIM S4 in Table 3). Those cleavage sites targeting the non-coding strand were cleaved only once by the CRISPR1-Cas system. Here, all CRISPR3 BIMs tested had a unique cleavage site three bases upstream of the PAM, whatever the orientation of the protospacer on the phage genome (coding and non-coding strand).

The identification of the CRISPR3-Cas cleavage site within the protospacer is also in agreement with the recent work of Sapranauskas *et al*. [16], who showed that mutations in the 25^th^ or 28^th^ nucleotide of the protospacers (i.e., positions -6 and -3 respectively from the PAM sequence) impair CRISPR3 interfering activity. Mutations at other positions, including those corresponding to the second cleavage site observed for the CRISPR1-Cas system, did not affect the plasmid interference ability of the CRISPR3-Cas system [16].

### Conclusion

Using the phage-host system 2972-DGCC7710 and our laboratory conditions, we showed that the acquisition stage of the CRISPR1-Cas system can be more active than the CRISPR3-Cas system. The presence of 32 spacers in the CRISPR1 locus of the wild-type phage-sensitive strain *S. thermophilus* DGG7710, compared to 12 spacers in the CRISPR3 locus, supports increased adaptation activity for the CRISPR1-Cas system. The reasons for such a discrepancy are unclear at this time. It is not due to the number of PAMs in the phage genome since more CRISPR3 PAM sequences are found in the genome of phage 2972 [12]. On the other hand, the interference conferred by both CRISPR loci was similar, as illustrated by comparable phage EOPs. We also showed that two CRISPR-Cas systems are compatible within one bacterial strain, as illustrated by the increased phage resistance (Table 3) and by the double cleavage of the invading dsDNA (Figure 2). Finally, both CRISPR-Cas type II systems target and cleave dsDNA phages at the same preferential site, located three nucleotides upstream of their respective PAMs.

The industrial strain *S. thermophilus* DGCC7710 is equipped with at least two active and compatible CRISPR-Cas systems to defend against, among others, virulent phages. Comparative analyses of some *S. thermophilus* genomes revealed very high similarity with only few divergent regions including the CRISPR-Cas systems [1], [18]–[21]. Additional studies found that CRISPR1 is ubiquitous in *S. thermophilus* strains whereas CRISPR3 is less frequent [13]. It appears that *S. thermophilus* recently emerged by a combination of loss-of-function and also horizontal gene transfer events that contributed to its adaptation to the milk environment [1], [19]–[21], which is contaminated with diverse populations of virulent phages [22]. It is tempting to speculate that the acquisition of multiple CRISPR-Cas systems provided a remarkable flexibility and helped this species to quickly adapt and thrive in the dairy environment.

## Materials and Methods

### Bacterial Strains, Phages, and Culture Conditions

*S. thermophilus* strains were grown in M17 broth supplemented with 0.5% lactose (LM17) at 37°C or 42°C. The spacer content of all *S. thermophilus* strains was always confirmed by sequencing of the CRISPR loci [23]. Phages were propagated in LM17 media containing 10 mM CaCl_2_ (LM17-CaCl_2_) as described elsewhere [5]. High-titer phage preparations (10^10^–10^11^ PFU mL^−1^) were obtained using PEG precipitation followed by chloroform purification [24]. The efficiency of plaquing (EOP) was determined as reported [12], with the exception that *S. thermophilus* strains had reached an optical density at 600 nm (OD_600_) of 0.6 before plating.

### Phage Infections

*S. thermophilus* DGCC7710 and derivatives were incubated at 42°C in 1 L of LM17-CaCl_2_ until they reached an OD_600_ of 0.6. The latent period of the virulent phage 2972 was previously determined to be 40±3 minutes (min) in *S. thermophilus* DGCC7710, with a maximum burst size of 190±33 phages released per infected cell [12], [25]. After removing a 100 mL sample of uninfected cells (NI), each bacterial culture was infected with purified phage 2972 at a multiplicity of infection of 5 and incubated at 42°C. Samples were taken after 45 min, centrifuged for 5 min at 16,000 g, and pellets were flash frozen and stored at −80°C until DNA extraction.

### DNA Extraction and Southern Hybridization

Total DNA extractions were performed as described elsewhere [26] with reported modifications [15]. Five micrograms of DNA from a phage-infected phage-resistant (BIM) of *S. thermophilus* and 1 µg of DNA from a phage-infected wild-type strain were digested with SpeI restriction enzyme (Roche) before migration on six replicated 0.8% agarose gel (one per probe). The DNA was then transferred onto positively charged nylon membrane by capillary blotting [27]. One nanogram of digested phage DNA was used as positive control for probe hybridizations. DNA probes were prepared with PCR DIG labeling mix (Roche) (Supplementary Table S1). Pre-hybridization, hybridization, washes and detection (CDP-star) were performed as recommended by Roche.

### Phage DNA Cleavage Site Determination

CRISPR-induced cleavage sites were determined as described previously [15]. Briefly, extracted DNA from infected BIMs were digested independently using different blunt restriction enzymes (DraI, EcoRV, MlyI, ScaI, and/or SspI) and visualized on an agarose gel, before being eluted and purified for ligation (Roche). Inverse PCR was done using primers listed in Supplementary Table S2, then sent for sequencing to determine the cleavage site.

## Supporting Information

Table S1 **A. Refer to** **Figure 2** **for the position of the protospacer and for restriction sites in the phage 2972 genome.** B. The position of the primer in the phage 2972 genome.
(DOCX)

Table S2 **A. Refer to** **Figure 2** **for the position of the protospacer and for restriction sites in the phage 2972 genome.** B. Primers used only for sequencing are specified (*). All other primers were used for both inverse PCR and sequencing. C. The position of the protospacer in the phage 2972 genome.
(DOCX)
