# Supplementary material for: Cleavage of Phage DNA by the Streptococcus thermophilus CRISPR3-Cas System
Source: PLoS One. 2012 Jul 20;7(7):e40913. doi: 10.1371/journal.pone.0040913 (PMC3401199; doi:10.1371/journal.pone.0040913)
Supplement: Table S1 — A. Refer to Figure 2 for the position of the protospacer and for restriction sites in the phage 2972 genome. B. The position of the primer in the phage 2972 genome. (DOCX) [file pone.0040913.s001.docx]

**TABLE S1. Primers and probes used for Southern blot assays.**

| **PCR amplified region^a^** | **Name** | **Sequence (5'⭢3')** | **Position^b^** |
| --- | --- | --- | --- |
| 3’-PS61 | JG-58 | TTGCAGAAGGCGCTATTTATGCCG | 2,417 - 2,440 |
|  | JG-59 | TCGTGCTGTTCTCTTTCCATCGGT | 2,866 - 2,843 |
| 5’-PS61 | Sonda94LF | GCATTGCTGGTAGCAGACTGG | 231 - 251 |
|  | Sonda94LR | ACCGCTTGTTCTATGGCTTC | 637 - 618 |
| 3’-PS78 | 138LF | GACAGACGAGAATGGCATGA | 27,885 - 27,904 |
|  | 138RR | AGTTCCTTCTGGTAGTCCCG | 28,128 - 28,109 |
| 5’-PS78 | 153LF | CAAAAACCAGTATGGACACCA | 26,645 - 26,665 |
|  | 153RR | TACTTCAATCCGCACCCATC | 26,872 - 26,853 |
| 3’-PS85 | 146LF | GATGTTGTGCAAGGCTCTGAC | 16,446 - 16,466 |
|  | JL-15 | GGTTTGCAGTCCAACTCGTA | 18,191 - 18,172 |
| 5’-PS85 | JG-60 | ATGTCAACGACACCAAGCCAAACG | 15,763 - 15,786 |
|  | JG-61 | TGTGTGTAACAAGTGGTGCGTTGC | 16,305 - 16,282 |
