# Supplementary material for: Cleavage of Phage DNA by the Streptococcus thermophilus CRISPR3-Cas System
Source: PLoS One. 2012 Jul 20;7(7):e40913. doi: 10.1371/journal.pone.0040913 (PMC3401199; doi:10.1371/journal.pone.0040913)
Supplement: Table S2 — A. Refer to Figure 2 for the position of the protospacer and for restriction sites in the phage 2972 genome. B. Primers used only for sequencing are specified (*). All other primers were used for both inverse PCR and sequencing. C. The position of the protospacer in the phage 2972 genome. (DOCX) [file pone.0040913.s002.docx]

**TABLE S2. Primers used for cleavage site determinations.**

| **PCR amplified region^a^** | **Name** | **Sequence (5' ⭢ 3')** | **Position^b^** |
| --- | --- | --- | --- |
| 3’-PS61 | 94LR | CCTGTAGCGGCATTTAGCTC | 1244 - 1225 |
|  | 94LF | CTCGTGTGGTTTGGGATAGC | 1905 - 1924 |
| 5’-PS61 | 94RR | CTCATGCCGATTCAACTTAGC | 2273 - 2253 |
|  | 94RF^*^ | TGTCATCGGTAGCACAGAGC | 2934 - 2953 |
|  | 94RRF | GCGATTAAGCCCGTTAGAGA | 3503 - 3522 |
| 3’-PS73 | 156LR | CAACGACCACGGCTATCAGAT | 22,329 - 22,309 |
|  | 156LF^*^ | GAACCCGGGCAGTATTACCT | 22,889 - 22,908 |
|  | 156LLF | GTATGCAGGATATGAAGGCAGC | 23,137 - 23,158 |
| 5’-PS73 | 70RR | ATTACTTTGGTGGCTGACGC | 23,497 - 23,478 |
|  | 138RF | GAAGCTCATCATATCAAAGCTAAAC | 28,412 - 28,436 |
|  | CL-27^*^ | TGGCACACCTATCCGATTAG | 28,489 - 28,508 |
| 3’-PS75 | 73RR | TGTTCGCCTTTAGCGTCCTC | 24,553 - 24,534 |
|  | 138RF | GAAGCTCATCATATCAAAGCTAAAC | 28,412 - 28,436 |
|  | CL-27^*^ | TGGCACACCTATCCGATTAG | 28,489 - 28,508 |
| 5’-PS75 | 73LR | CAAGCCTACAAGCTCTTGCA | 23,940 - 23,921 |
|  | 73LLR | GATATTGAGCAAAAGTCGTAGTCG | 21,533 - 21,510 |
|  | 73LF | AAAATCAGCAGCAAATGGCT | 24,208 - 24,227 |
|  | 73LLF | TGGTTAGCGGTATGATGTGG | 24,112 - 24,131 |
| 3’-PS77 | 153LR | GCAAAACTTCCTTGAGAACCG | 26,441 - 26,421 |
|  | 153LF | CAAAAACCAGTATGGACACCA | 26,645 - 26,665 |
| 5’-PS77 | 153RR | TACTTCAATCCGCACCCATC | 26,872 - 26,853 |
|  | 153RF | CAACAATTAGGGAGGGTAGGAA | 26,938 - 26,959 |
| 3’-PS78 | 153LF | CAAAAACCAGTATGGACACCA | 26,645 - 26,665 |
|  | 153RR | TACTTCAATCCGCACCCATC | 26,872 - 26,853 |
| 5’-PS78 | 138LF | GACAGACGAGAATGGCATGA | 27,885 - 27,904 |
|  | 138RR | AGTTCCTTCTGGTAGTCCCG | 28,128 - 28,109 |
| 3’-PS79 | 138RR | AGTTCCTTCTGGTAGTCCCG | 28,128 - 28,109 |
|  | CL-27 | TGGCACACCTATCCGATTAG | 28,489 - 28,508 |
| 5’-PS79 | 153LR | GCAAAACTTCCTTGAGAACCG | 26,441 - 26,421 |
|  | 138LF | GACAGACGAGAATGGCATGA | 27,885 - 27,904 |
| 3’-PS81/PS82 | 106LR | CGTCACCGTAAATCACACGT | 28,706 - 28,687 |
|  | 106LF | GTTTGCCATGAGGGCTTTAA | 28,996 - 29,015 |
| 5’-PS81/PS82 | 106RR | AGCACTCCAAAAAGTTGCGT | 29,197 - 29,178 |
|  | 106RF | ACCAGTTGGAAGGAAAAGCTC | 29,733 - 29,753 |
